# Spatial and temporal inequalities in malaria incidence and mortality among children aged 0–4 years in Nigeria: a subnational analysis, 2010–2019

**DOI:** 10.1186/s12936-026-05809-z

**Published:** 2026-02-02

**Authors:** Tolutope Adebimpe Oso, Olalekan John Okesanya, Uthman Okikiola Adebayo, Oluwatobi Babajide Ayelaagbe, Mohamed Mustaf Ahmed, Khalifat Boluwatife Obadeyi, Gilbert Eshun, Olanrewaju Mustapha Saliu, Don Eliseo Lucero-Prisno

**Affiliations:** 1 Department of Medical Laboratory Science, Neuropsychiatric Hospital, Aro, Abeokuta, Nigeria; 2 Department of Medical Laboratory Science, McPherson University, Seriki Sotayo, Ogun State Nigeria; 3 Faculty of Medicine, Department of Public Health and Maritime Transport, University of Thessaly, Volos, Greece; 4 Department of Medical Laboratory Science, College of Basic Health Sciences, Achievers University, Owo, Ondo Nigeria; 5 Faculty of Medicine and Health Sciences, SIMAD University, Mogadishu, Somalia; 6 Department of Research and Innovations, eHealth Somalia, Mogadishu, Somalia; 7 The Royal (Dick) School of Veterinary Studies and the Roslin Institute, University of Edinburgh, Midlothian, UK; 8 Department of Medicine and Surgery, Al-Aqiq General Hospital, Al-Aqiq, Al-Baha Province Saudi Arabia; 9 Faculty of Basic and Applied Biological Science, Department of Public Health, Ahmadu Bello University, Zaria, Nigeria; 10 Department of Global Health and Development, London School of Hygiene and Tropical Medicine, London, UK; 11 Center for University Research, University of Makati, Makati City, Philippines

**Keywords:** Malaria incidence, Malaria mortality, Under-five malaria, Plasmodium falciparum, Childhood malaria, Nigeria

## Abstract

**Background:**

Malaria remains a leading cause of morbidity and mortality among children under five in Nigeria, with pronounced subnational disparities. This study analyzed the temporal and spatial inequalities in malaria incidence and mortality among children aged 0–4 years across Nigeria’s 36 states and the Federal Capital Territory from 2010 to 2019.

**Methods:**

Estimates from the Institute for Health Metrics and Evaluation (IHME) were analyzed using the WHO Health Equity Assessment Toolkit (HEAT). Subnational inequalities were quantified using five metrics: coefficient of variation (COV), difference (D), ratio (R), population-attributable risk (PAR), and population-attributable fraction (PAF).

**Results:**

From 2010 to 2019, national malaria incidence declined from 103 to 74.5 cases (27.7% reduction), while mortality fell from 477.3 to 237.6 deaths per 100,000 (50.2% reduction). However, progress was uneven. Northern states such as Zamfara, Kano, and Katsina had the highest baseline burden in 2010 (incidence > 150 per 1000; mortality > 700 per 100,000) and, despite declines, remained among the most affected in 2019. Southern states including Lagos, Delta, and Anambra consistently recorded lower burdens (incidence < 60 per 1000; mortality < 300 per 100,000). Subnational inequality narrowed over time, with incidence COV peaking at 46.3% in 2013 before falling to 28.1% in 2019, and mortality COV declining from a 2013 peak of 42.9% to 22.3% in 2019.

**Conclusion:**

Nigeria’s malaria incidence and mortality among under-five children have decreased, but subnational disparities persist, particularly in northern states, although a reduction in D and R values indicates modest progress in equity, necessitating geographically targeted interventions.

## Introduction

Malaria remains one of the world’s most devastating infectious diseases and continues to pose a major global public health challenge with a disproportionately high burden in sub-Saharan Africa. Despite significant investments in malaria control and elimination strategies over the past two decades, malaria has occurred in many high-burden countries [1]. According to the World Health Organization (WHO), an estimated 282 million malaria cases and 610,000 malaria-related deaths occurred globally in 2024, representing a slight increase from the previous year. The burden of malaria remains disproportionately concentrated in the WHO African Region, where young children under five years of age continue to be the most vulnerable group and account for the majority of malaria-related deaths [2]. According to WHO estimates, Nigeria continues to bear the highest malaria burden worldwide, contributing to a substantial proportion of global malaria cases and deaths [2]. This overwhelming burden underscores the persistent and complex challenges faced by malaria control efforts in Nigeria. The high incidence and mortality rates are driven by a combination of environmental factors, such as diverse ecological zones that support year-round transmission, epidemiological pressures, socioeconomic disparities, and health system limitations that hinder timely diagnosis, treatment, and prevention [3].

This study used the Social Determinants of Health (SDH) framework to analyze these complex factors. The framework demonstrates that health outcomes result from biological and clinical elements, as well as the birth environment and people’s life experiences. The spatial heterogeneity of malaria transmission in Nigeria is driven by diverse ecological zones, ranging from mangrove swamps and rainforests in the south to savannahs and semi-arid regions in the north. These ecological variations influence the seasonality and intensity of malaria transmission, vector density, and species composition [4, 5]. Similarly, inequalities in access to healthcare, socioeconomic status, maternal education, and geographic accessibility create localized pockets of high malaria burden that are often masked by the national averages [6]. The presence of internally displaced populations due to conflict, particularly in the northeastern and north-central states, has further exacerbated the vulnerability of children to malaria in these regions [7].

Although Nigeria has made strides in scaling up malaria interventions, including the distribution of insecticide-treated nets (ITNs), intermittent preventive treatment in pregnancy (IPTp), and the introduction of artemisinin-based combination therapies (ACTs), disparities persist in the accessibility and effectiveness of these interventions at the sub-national level [8]. For instance, the 2021 Nigeria Malaria Indicator Survey (NMIS) revealed considerable interstate differences in ITN ownership and usage, access to prompt treatment, and IPTp uptake [9]. Nationally, 56% of households owned at least one ITN, while only 41% of children under five slept under an ITN the previous night, indicating a pronounced ownership-use gap in the country. ITN ownership was generally higher in the northern zones, whereas net use among children under five years was lowest in the South West and South South (below 25%) and highest in the North East and North West (exceeding 50%). These patterns are consistent with evidence showing lower odds of ITN ownership among women of reproductive age in several southern zones than in northern zones [9, 10]. Childhood mortality due to malaria in Nigeria is not only a function of transmission intensity but also of health system responsiveness, care-seeking behavior, and coexisting conditions such as malnutrition and anaemia. Children under five years of age are particularly susceptible to severe malaria due to their underdeveloped immune systems, making prompt diagnosis and effective treatment critical for survival [11]. However, many states in Nigeria still report unacceptably high child mortality rates due to malaria, reflecting systemic failures in timely access to life-saving interventions.

Moreover, although progress has been made, surveillance data still suffer from underreporting and incomplete data representation, particularly in rural and hard-to-reach areas, and variable data quality arising from weaknesses in routine health information systems [12]. While several national surveys and modelling efforts have provided estimates of the malaria burden in Nigeria, there is a scarcity of longitudinal analyses exploring trends in malaria incidence and mortality at the subnational level over extended periods. Most existing studies either focus on malaria prevalence rather than incidence and mortality or lack the spatial resolution needed to inform targeted interventions [13]. Given the highly focal nature of malaria transmission in Nigeria, understanding where and how the burden persists or changes over time is essential for the effective allocation of resources, intervention prioritization, and policy planning.

The WHO’s High Burden to High Impact (HBHI) initiative emphasizes the importance of subnational data use and stratification to guide malaria response strategies, especially in high-burden countries such as Nigeria [14]. Consistent with this call, the National Malaria Strategic Plan (2021–2025) outlines the need for data-driven, state-level malaria programming to reduce malaria morbidity and mortality by 50% by 2025 [15]. A thorough analysis of subnational malaria trends can offer critical insights into where progress has been made, where it has stalled, and where intensified efforts are urgently needed to eliminate malaria in these regions. This study aimed to examine the temporal trends in malaria incidence and mortality among children aged 0–4 years across Nigeria’s 36 states and the Federal Capital Territory (FCT) from 2010 to 2019. Specifically, we assessed spatial clustering and the magnitude of subnational inequalities in these outcomes, identified persistent and emerging high-burden hotspots, and explored their association with the coverage of key malaria control interventions and selected sociodemographic indicators. By mapping these patterns and unpacking their drivers, this study aims to provide policymakers, public health practitioners, and funding agencies with actionable evidence to support evidence-based decision-making, guide equitable and efficient targeting of malaria control interventions, and inform subnational malaria programming in Nigeria.

## Methods

### Study background and data sources

This study utilized annual geospatial estimates produced by the Institute for Health Metrics and Evaluation (IHME) in collaboration with the Malaria Atlas Project to assess malaria incidence and mortality across the 36 states of Nigeria and the FCT among children aged 0–4 years, covering the period from 2010 to 2019. These estimates were derived from geographically referenced data collected through large-scale national household surveys and routine, malaria surveillance. The primary data sources included the Demographic and Health Survey (DHS), Malaria Indicator Survey (MIS), and other country-specific surveys, providing comprehensive insights into the trends and disparities in the malaria burden across the country’s diverse subnational regions [16].

The primary data used in this study were derived from nationally representative household surveys, including the DHS and MIS, which employed distinct sampling strategies. The DHS uses a stratified, multi-stage cluster sampling design with population-proportional allocation across extended survey periods, resulting in substantial seasonal mixing that may limit its suitability for capturing malaria transmission intensity. In contrast, the MIS is specifically designed to assess the malaria burden and intervention coverage, with clusters allocated to better capture malaria-risk heterogeneity, often oversampling rural and high-transmission areas, and fieldwork conducted during peak malaria transmission seasons. These differences imply that DHS and MIS estimates are not directly interchangeable, particularly for parasite prevalence and seasonally sensitive indicators, and may influence the spatial and temporal patterns observed in this study.

This approach captured variations across state-level administrative units (the 36 states of Nigeria and the Federal Capital Territory), urban and rural settings, and key demographic subgroups within the under-five population (0–4 years), thereby reflecting the complex epidemiological landscape of Nigeria. Sampling within the primary data sources followed the survey-specific designs of the DHS and MIS, which account for geographic heterogeneity and regional variations in malaria transmission. The characteristics of each survey’s sampling framework were considered when interpreting malaria estimates. These data inputs were subsequently integrated into the IHME spatiotemporal geostatistical modelling framework to generate predictive subnational malaria estimates at a high spatial resolution. All estimates are reported with corresponding 95% uncertainty intervals to capture the uncertainty arising from the survey design, measurement error, and uneven data availability across regions. This approach facilitates informed, evidence-based decision-making and supports targeted malaria control strategies while transparently communicating the associated uncertainties.

The 2010–2019 IHME malaria dataset was accessed via the WHO Health Equity Assessment Toolkit (HEAT) online platform, which facilitates the immediate exploration and analysis of health inequality data [17]. The study design and reporting process were informed by the Strengthening the Reporting of Observational Studies in Epidemiology (STROBE) guidelines, which ensured methodological rigor and transparency. Relevant elements applied include clearly stating the study objectives, describing the data sources, specifying the analytic methods, and presenting the results with appropriate uncertainty intervals. We note that certain components, such as participant recruitment, direct bias mitigation strategies, and handling of missing primary data, do not directly apply because this study relies on secondary, modelled data derived from nationally representative surveys and geospatial models [18].

### Setting description

Nigeria is divided into 36 states and the FCT, grouped into six geopolitical zones for administrative and policy purposes: North Central, North East, North West, South East, South South, and South West. Each zone comprises multiple states with shared geographic, sociocultural, and economic characteristics. While our analyses focused on the state-level malaria burden among children aged 0–4 years, the geopolitical zone framework provides context for regional variation and allows readers to understand the broader subnational distribution patterns observed in this study.

### Inequality variable

The outcome variables in this study were malaria incidence and mortality. Malaria incidence is reported as the number of new cases per 1000 population per year, and malaria mortality is reported as the number of deaths per 100,000 population per year. Both outcomes were derived from the IHME’s modelled estimates, which integrated survey data and geospatial covariates, with 95% uncertainty intervals reflecting the precision of the estimates. These indicators were derived from geospatial modelling based on national household survey data and surveillance systems and reflect the malaria burden at the state level. The subnational states were classified into Abia, Abuja (FCT), Adamawa, Akwa Ibom, Anambra, Bauchi, Bayelsa, Benue, Borno, Cross River, Delta, Ebonyi, Edo, Ekiti, Enugu, Gombe, Imo, Jigawa, Kaduna, Kano, Katsina, Kebbi, Kogi, Kwara, Lagos, Nasarawa, Niger, Ogun, Ondo, Osun, Oyo, Plateau, Rivers, Sokoto, Taraba, Yobe, and Zamfara, covering all 36 states of Nigeria and FCT.

### Statistical tool

The WHO HEAT online software was used for the analyses, providing an interactive and customizable platform for interpreting and reporting health inequality. HEAT was chosen over HEAT Plus because it includes preloaded survey data for Nigeria, enabling the standardized calculation and visualization of health inequality measures without the need to upload custom datasets. The toolkit uses various health and social indicators to analyze health disparities, particularly in LMICs such as Nigeria. Health inequality monitoring, including the statistics package of WHO HEAT, illustrates the process through examples and promotes the integration of health inequality monitoring within health information systems in these countries [19]. Five measures were used to assess inequality: the coefficient of variation (COV), difference (D), ratio (R), population-attributable fraction (PAF), and population-attributable risk (PAR). These measures were selected because they provide complementary perspectives on income inequality. COV captures relative dispersion, D and R quantify the absolute and relative differences between subgroups, and PAF and PAR estimate the contribution of subgroup disparities to population-level outcomes. As the WHO HEAT does not include statistical testing or uncertainty estimates, it limits the interpretability of differences in inequality measures over time.

### Coefficient of variation (COV)

The COV is a relative measure of inequality that quantifies the degree of variability in malaria incidence or mortality rates across subnational regions. It expresses the extent of dispersion around the mean, providing insight into how unevenly the malaria burden is distributed geographically

$${\text{Formula }}\left( {{\mathrm{Incidence}}} \right):{\mathrm{COV}}_{{{\mathrm{incidence}}}} = \frac{{\sigma_{{{\mathrm{incidence}}}} }}{{\mu_{{{\mathrm{incidence}}}} }} \times 100$$

$${\text{Formula }}\left( {{\mathrm{Mortality}}} \right):{\mathrm{COV}}_{{{\mathrm{mortality}}}} = \frac{{\sigma_{{{\mathrm{mortality}}}} }}{{\mu_{{{\mathrm{mortality}}}} }} \times 100$$

where:

σ = Standard deviation of malaria incidence or mortality rates across all subnational regions.

μ = Mean (average) malaria incidence or mortality rate.

COV is expressed as a percentage (%).

A higher COV indicates greater relative inequality in the malaria burden across the states. A COV of 0% indicates no variation; all regions have the same rate.

### Difference (D)

Difference is an absolute measure of inequality that quantifies the gap in malaria incidence or mortality rates between the most advantaged and disadvantaged subnational states. In this context, “disadvantaged” and “advantaged” refer to the states with the highest and lowest rates, respectively, for the outcome of interest.

$${\text{Formula }}\left( {{\mathrm{Incidence}}} \right):D_{{{\mathrm{incidence}}}} = {\text{Incidence rate}}_{{\text{most disadvantaged}}} - {\text{Incidence rate}}_{{\text{most advantaged}}}$$

$${\text{Formula }}\left( {{\mathrm{Mortality}}} \right):D_{{{\mathrm{mortality}}}} = {\text{Mortality rate}}_{{\text{most disadvantaged}}} - {\text{Mortality rate}}_{{\text{most advantaged}}}$$

A higher D value indicates a greater absolute disparity between the state. A D value of zero indicates equality of distribution.

### Ratio (R)

A ratio is a relative measure that compares the malaria incidence or mortality rate in the most disadvantaged subnational states with that in the most advantaged state. Disadvantaged and advantaged groups refer to states with the highest and lowest rates, respectively, for the outcome of interest.

$${\text{Formula }}\left( {{\mathrm{Incidence}}} \right):R_{{{\mathrm{incidence}}}} = \frac{{{\text{Incidence rate}}_{{\text{most disadvantaged}}} }}{{{\text{Incidence rate}}_{{\text{most advantaged}}} }}$$

$${\text{Formula }}\left( {{\mathrm{Mortality}}} \right):R_{{{\mathrm{mortality}}}} = \frac{{{\text{Mortality rate}}_{{\text{most disadvantaged}}} }}{{{\text{Mortality rate}}_{{\text{most advantaged}}} }}$$

A ratio greater than 1 indicates that the disadvantaged subnational state experiences higher malaria incidence or mortality rates than the advantaged group. A ratio of one indicated parity.

### Population attributable risk (PAR)

PAR is an absolute measure that estimates the difference between the average malaria incidence or mortality rate in the total population and the rate observed in the most advantaged subnational states. Here, “advantaged” refers to the state with the highest rate. It reflects how much the overall incidence or mortality could be reduced if all regions had the same rates as the most advantaged subnational states.

$${\text{Formula }}\left( {{\mathrm{Incidence}}} \right):{\mathrm{PAR}}_{{{\mathrm{incidence}}}} = {\upmu }_{{{\mathrm{incidence}}}} - {\text{Incidence rate}}_{{\text{most advantaged}}}$$

$${\text{Formula }}\left( {{\mathrm{Mortality}}} \right):{\mathrm{PAR}}_{{{\mathrm{mortality}}}} = {\upmu }_{{{\mathrm{mortality}}}} - {\text{Mortality rate}}_{{\text{most advantaged}}}$$

where μ (incidence) and μ(mortality) are the average incidence and mortality rates across all subnational states. Negative PAR values may arise when the national average is lower than the estimate for the reference (most advantaged) state. In the context of adverse health indicators, such as malaria incidence and mortality, negative PAR values are expected and indicate the potential reduction in the national burden if all states achieve the level observed in the most advantaged state. Thus, the sign of PAR reflects the direction of inequality, whereas its magnitude represents the absolute extent of the avoidable disease burden.

### Population attributable fraction (PAF)

The PAF is a relative measure that expresses the proportion of total malaria incidence or mortality in the population that can be attributed to inequalities. It represents the percentage reduction in the overall malaria burden that could be achieved if all states had the same rates as the most advantaged group.

$${\text{Formula }}\left( {{\mathrm{Incidence}}} \right):{\mathrm{PAF}}_{{{\mathrm{incidence}}}} = \frac{{{\upmu }_{{{\mathrm{incidence}}}} }}{{{\mathrm{PAR}}_{{{\mathrm{incidence}}}} }} \times 100$$

$${\text{Formula }}\left( {{\mathrm{Mortality}}} \right):{\mathrm{PAF}}_{{{\mathrm{mortality}}}} = \frac{{{\upmu }_{{{\mathrm{mortality}}}} }}{{{\mathrm{PAR}}_{{{\mathrm{mortality}}}} }} \times 100$$

A positive PAF indicates that inequality contributes to a higher national malaria burden, and addressing these disparities could lead to substantial reductions in the burden.

## Results

**Fig. 1:**
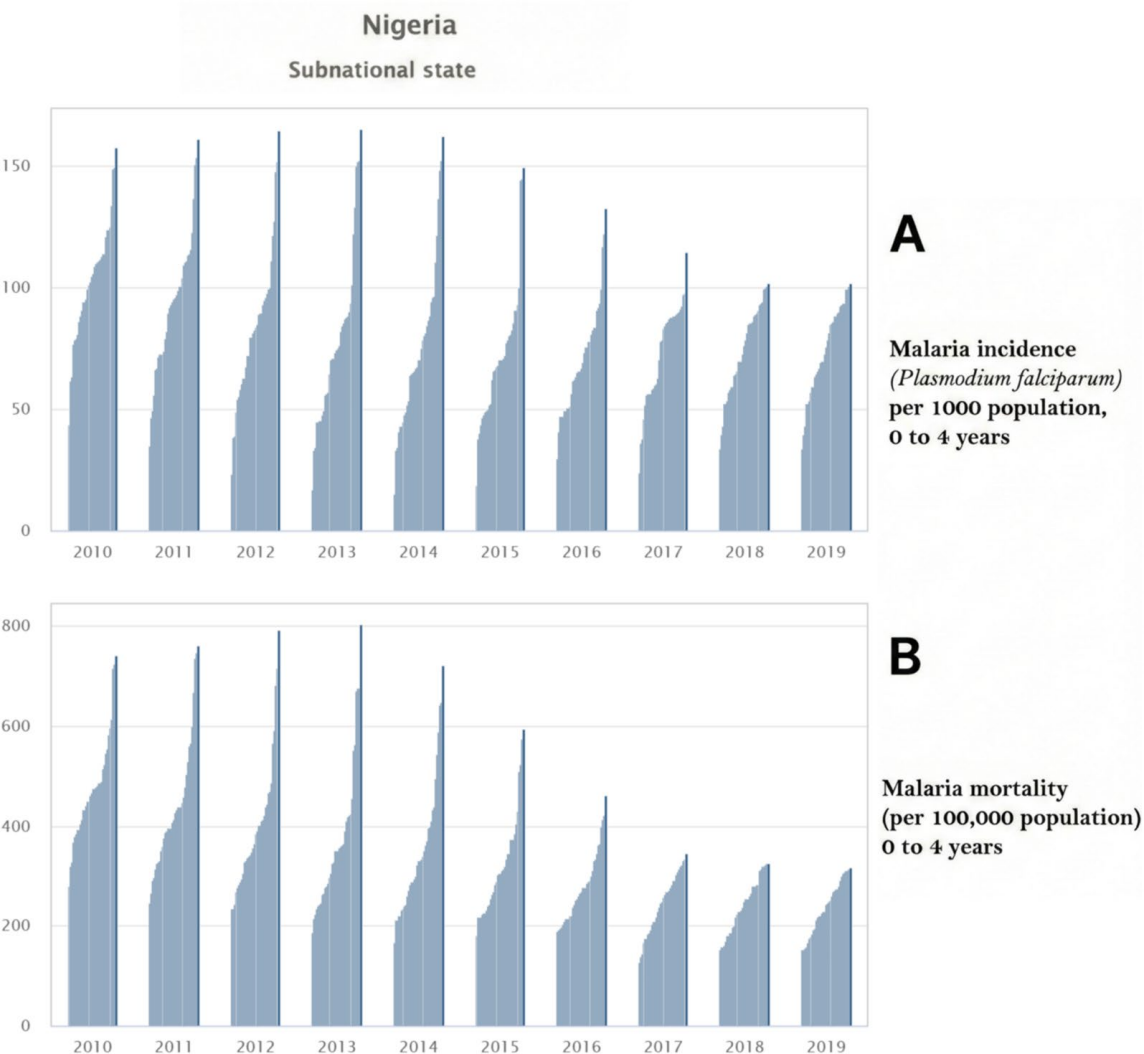
Subnational distribution of malaria burden among children aged 0–4 years in Nigeria, 2010–2019. **A** Subnational distribution of *Plasmodium falciparum* malaria incidence rates among children aged 0–4 years in Nigeria, 2010–2019. **B** Subnational distribution of malaria mortality rates (per 100,000 population) among children aged 0–4 years in Nigeria, 2010–2019. X-axis (both panels): Year (2010–2019). Y-axis **A**: Malaria incidence rate. Y-axis **B**: Malaria mortality rate per 100,000 population. Bars represent Nigerian states ranked within each year from lowest to highest malaria burden. Estimates are shown for children aged 0–4 years. Data were obtained from nationally comparable modeled estimates used for subnational inequality analysis

**Fig. 2:**
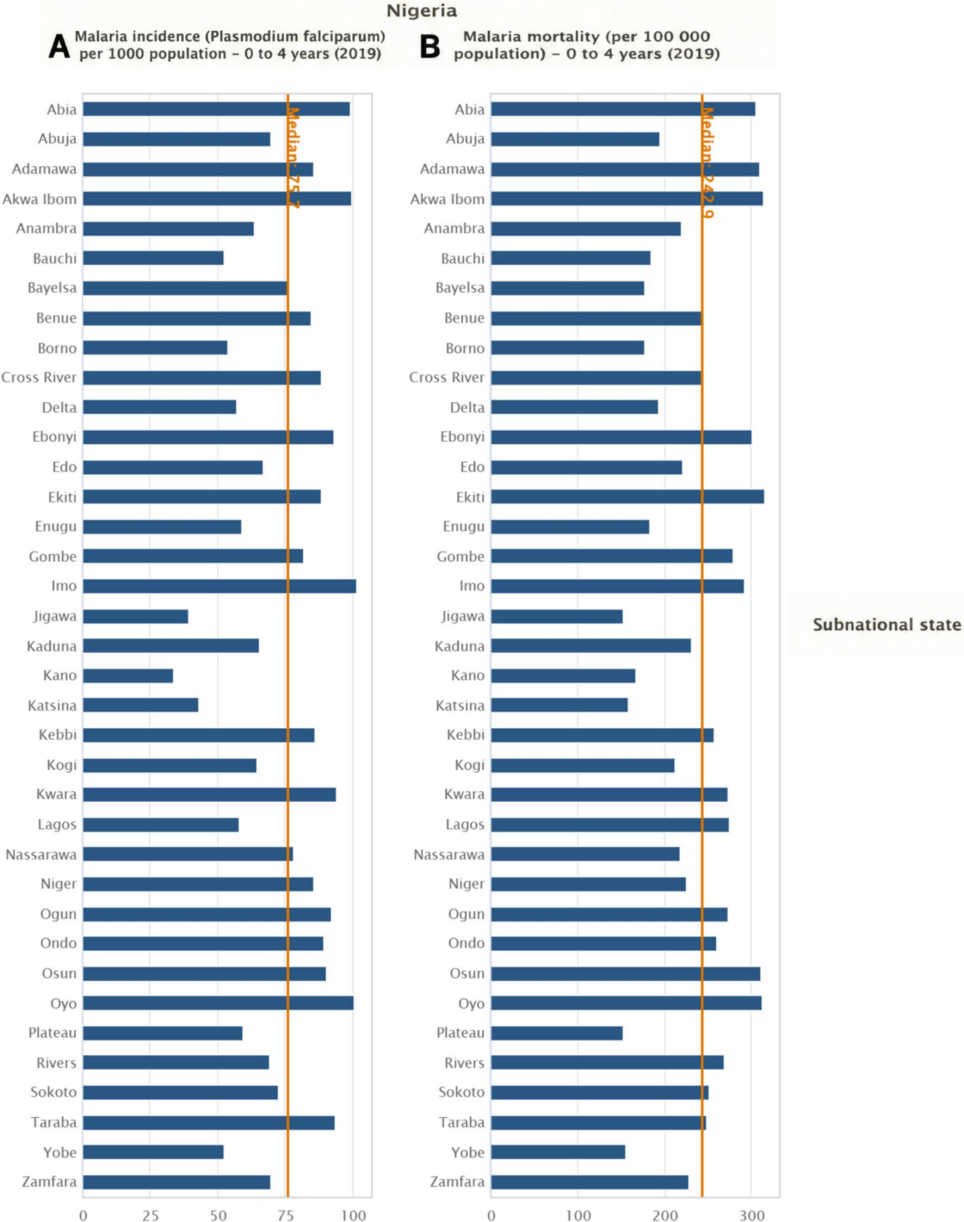
Subnational variation in malaria incidence and mortality among children aged 0–4 years in Nigeria, 2019. **A** State-level malaria incidence (*Plasmodium falciparum*, %) among children aged 0–4 years in Nigeria, 2019. **B** State-level malaria mortality (per 100,000 population) among children aged 0–4 years in Nigeria, 2019. X-axis **A**: Malaria incidence (%). X-axis **B**: Malaria mortality rate per 100,000 population. Y-axis (both panels): Nigerian states. Bars represent state-level estimates for children aged 0–4 years. The vertical reference line indicates the national median value for each indicator. Estimates were generated using the Health Equity Assessment Toolkit (HEAT) based on data from the WHO Health Inequality Monitor (2024 update). These data originate from external published sources and do not represent official WHO estimates

This retrospective study of secondary, modelled data examined the pattern of subnational inequalities in malaria (*Plasmodium falciparum)* incidence and mortality rate among children aged 0–4 years across Nigeria’s 36 states and the Federal Capital Territory (FCT) over a decade (2010–2019). The results demonstrated marked subnational differences in the malaria burden between states, highlighting persistent inequalities in the disease outcomes. Figure 1 illustrates the trends in malaria incidence and mortality among children aged (0–4 years) in Nigeria by Subnational Region (2010–2019), and Fig. 2 shows the malaria incidence and mortality among children aged (0–4 years) in Nigeria by Subnational Region in 2019.

### Temporal trends in malaria incidence (Plasmodium falciparum)

Table 1 shows the malaria incidence (per 1000) of *Plasmodium falciparum and* mortality rate (per 100,000 population) among children aged 0–4 years across the 36 states in Nigeria and the Federal Capital Territory (FCT), Abuja, from 2010 to 2019. Malaria incidence at the national level showed an overall decline, although the rate and extent of the reduction differed among the states. In 2010, the annual malaria incidence among children aged 0–4 years ranged from 43.9 cases per 1000 children in Lagos State to 157.3 cases per 1000 children in Zamfara State, reflecting the average number of malaria infections per child per year. In some states in Northern Nigeria, such as Katsina, Kebbi, Kano, and Sokoto, incidence levels consistently exceeded 120 cases per 1000 in the early years of the study period (2010–2013). A considerable reduction in incidence was observed in many of these high-burden states in 2019 (Fig. 2). For example, Kano and Zamfara States experienced a decline in malaria incidence from 133.2 cases per 1000 and 165.0 cases per 1000 in 2013 to 33.5 cases per 1000 and 69.9 cases per 1000 in 2019, respectively.

**Table 1:** Temporal trends in malaria incidence (per 1000 population)

State	2010	2011	2012	2013	2014	2015	2016	2017	2018	2019
Abia	94.3	72.4	55.4	45.5	43.3	47.9	63.4	83.6	99.7	99.3
Abuja (FCT)	86.3	74.0	63.0	56.0	51.8	49.0	50.7	58.5	69.8	69.5
Adamawa	90.6	81.8	79.5	82.2	86.8	90.9	93.4	92.2	85.5	85.4
Akwa Ibom	88.3	66.7	51.9	45.3	45.4	51.6	66.7	85.3	99.8	99.4
Anambra	59.4	46.5	38.1	34.4	34.6	37.9	46.9	58.2	63.8	63.7
Bauchi	121.2	111.2	100.1	90.1	80.3	67.4	54.3	48.9	52.3	52.4
Bayelsa	78.9	72.7	67.2	64.3	64.2	65.9	73.1	78.3	76.1	75.7
Benue	123.8	113.3	99.3	87.9	81.5	78.4	82.6	87.1	84.9	84.6
Borno	80.9	71.2	63.1	56.8	53.4	50.1	49.7	51.9	53.7	53.7
Cross River	109.8	94.2	79.6	70.7	67.2	67.8	75.4	84.3	88.6	88.3
Delta	61.8	55.6	48.9	44.7	42.9	43.4	49.2	56.3	57.0	56.9
Ebonyi	123.7	109.3	94.8	84.1	78.7	77.3	83.6	90.9	93.2	92.9
Edo	101.1	93.3	81.2	70.7	64.8	62.3	65.9	70.2	67.2	67.0
Ekiti	110.4	98.9	84.4	74.4	70.6	72.2	80.7	87.9	88.7	88.2
Enugu	78.4	66.1	53.9	44.7	40.6	40.4	46.9	56.6	59.3	59.1
Gombe	99.2	89.7	85.2	85.3	88.2	90.7	91.6	88.4	81.5	81.6
Imo	95.2	73.9	58.2	49.3	47.9	52.5	69.0	88.9	101.7	101.5
Jigawa	111.0	115.8	121.8	122.0	110.5	82.6	50.7	35.9	39.5	39.5
Kaduna	101.5	95.7	95.7	97.8	94.0	80.9	62.5	55.6	65.7	65.6
Kano	106.0	114.0	127.2	133.2	121.5	85.3	40.9	24.1	33.5	33.5
Katsina	148.6	153.8	156.7	152.5	136.6	99.9	56.6	37.8	42.9	42.9
Kebbi	149.7	150.3	151.9	149.8	148.5	144.4	132.3	114.5	85.9	86.0
Kogi	108.7	92.1	71.9	56.5	48.6	46.3	51.1	59.7	64.8	64.4
Kwara	124.8	110.5	89.3	73.1	66.9	67.9	77.9	90.4	94.4	93.9
Lagos	43.9	34.7	23.4	16.6	15.3	18.5	29.4	45.8	58.3	58.1
Nasarawa	102.2	95.1	89.6	86.9	84.4	80.4	77.9	77.9	78.5	78.2
Niger	133.8	122.6	110.9	101.3	96.1	93.0	90.9	89.5	85.6	85.6
Ogun	94.2	79.1	60.3	47.5	44.6	49.5	64.9	83.2	92.4	92.2
Ondo	113.8	104.0	89.1	76.1	70.5	71.1	79.7	88.4	89.5	89.3
Osun	112.7	100.6	83.6	70.4	64.9	66.2	75.3	86.1	90.8	90.4
Oyo	114.1	100.9	82.7	69.2	65.7	70.4	83.9	97.1	100.7	100.5
Plateau	76.6	72.5	72.2	74.9	74.8	70.4	65.7	62.7	59.6	59.3
Rivers	63.2	49.5	38.7	33.3	33.1	36.9	47.2	60.6	69.5	69.3
Sokoto	128.1	136.4	147.4	151.9	152.1	144.7	122.4	97.8	72.5	72.6
Taraba	104.7	96.9	92.9	93.7	96.3	98.4	99.5	97.9	93.4	93.4
Yobe	111.8	105.1	97.6	88.4	80.3	70.2	61.4	56.3	52.4	52.5
Zamfara	157.3	161.2	164.8	165.0	161.9	149.6	116.8	87.9	69.8	69.9

Despite this progress, these states remain among those with the highest burdens in the country. In contrast, the Southern States generally reported lower malaria incidence rates throughout the study period. Delta, Anambra, Lagos, and Rivers States consistently recorded malaria incidence levels below the national average for the study period. Delta State showed a relatively stable decline from 61.8 cases per 1000 in 2010 to 56.9 cases per 1000 in 2019. Notably, Lagos State maintained one of the lowest malaria incidence levels throughout, with values declining to 58.1 cases per 1000 by 2019, despite a slight rebound after a low point in 2014 (15.3 cases per 1000). Some States showed more dynamic patterns; for instance, Imo State recorded a notable instability in the incidence rate, decreasing from 95.2 cases per 1000 in 2010 to 47.9 cases per 1000 in 2014, before increasing to 101.5 cases per 1000 by 2019. A similar late surge was observed in Akwa Ibom, with malaria incidence declining from 88.3 cases per 1000 in 2010 to 45.4 cases per 1000 in 2014, before increasing sharply to 99.4 cases per 1000 by 2019 (Table 1).

### Temporal trends in malaria mortality (per 100,000 population)

The malaria mortality rate exhibited a temporal trend broadly like that of malaria incidence, with the highest mortality rates mostly concentrated in the Northern States (Table 2). In 2010, malaria mortality among children aged 0–4 years ranged from 279.8 deaths per 100,000 population in Bayelsa to 739.9 deaths per 100,000 population in Katsina. Other states with notably high mortality rates included Zamfara and Kano, with 723.6 and 715.3 deaths per 100,000 population, respectively. Marked subnational differences in malaria mortality were observed across high-burden states over the past decade (2010–2019). For example, Kano State reduced its mortality rate from 715.3 in 2010 to 166.8 deaths per 100,000 in 2019. Zamfara State experienced a reduction in malaria mortality from 723.6 to 227.9 deaths per 100,000 during the study period. Similarly, Katsina State recorded a decline in mortality from 739.9 to 158.6 deaths per 100,000 people over the same period. These findings highlight marked progress in malaria control within the high-burden northern states, although mortality levels in these states remain relatively high compared with many southern states, reflecting persistent state-level subnational inequalities rather than a simple regional dichotomy. Malaria mortality patterns over the decade can be grouped into three categories: southern states with low, stable mortality, high-burden states with substantial improvements, and states exhibiting fluctuating trends.

1. Southern states with low, stable mortality

**Table 2:** Temporal trends in malaria mortality (per 100,000 population)

State	2010	2011	2012	2013	2014	2015	2016	2017	2018	2019
Abia	482.1	395.1	328.2	280.2	260.7	264.0	278.3	290.7	313.7	305.0
Abuja (FCT)	367.9	328.5	289.7	262.9	239.2	223.3	196.2	183.7	201.3	195.5
Adamawa	458.9	426.6	410.1	421.7	431.7	437.7	399.7	345.9	319.2	309.5
Akwa Ibom	477.9	389.9	330.4	295.9	287.6	291.4	298.9	303.9	325.5	313.5
Anambra	319.5	266.6	233.5	214.6	212.0	216.6	219.8	216.5	224.9	220.1
Bauchi	604.1	559.9	485.6	428.0	371.4	303.3	220.7	174.9	186.1	184.2
Bayelsa	279.8	247.1	235.2	224.7	220.5	217.5	209.9	191.1	182.4	177.5
Benue	467.4	438.6	391.3	356.4	331.2	312.6	290.7	262.4	253.4	246.9
Borno	405.9	358.6	305.2	264.6	240.8	218.8	192.8	175.9	180.3	176.7
Cross River	440.2	387.0	338.3	304.3	284.1	269.6	259.0	245.2	249.6	242.9
Delta	328.8	290.5	268.3	247.0	233.2	225.5	216.1	203.4	198.9	192.9
Ebonyi	597.3	530.1	468.8	424.7	395.3	372.8	345.2	313.9	310.6	300.7
Edo	475.1	433.2	402.9	361.4	331.5	305.5	277.5	248.4	229.9	221.7
Ekiti	555.6	503.0	467.0	419.2	398.9	383.7	363.7	334.7	325.8	315.7
Enugu	378.9	326.2	278.4	234.8	211.3	198.0	190.8	185.5	187.8	183.1
Gombe	485.9	450.3	420.3	410.7	409.5	403.9	362.5	307.7	282.6	279.1
Imo	474.3	396.6	346.7	305.1	288.8	281.7	287.3	289.9	300.7	292.6
Jigawa	546.4	566.6	566.1	551.7	493.9	372.3	215.7	137.8	153.5	152.9
Kaduna	460.0	447.1	443.1	455.9	437.1	372.2	269.6	209.5	237.6	230.5
Kano	715.3	760.1	793.3	802.9	721.1	522.9	243.5	126.0	170.3	166.8
Katsina	739.9	747.1	725.3	677.2	588.5	430.6	237.1	144.7	161.7	158.6
Kebbi	581.7	600.9	591.1	563.6	544.0	508.4	422.1	331.7	261.3	257.4
Kogi	448.8	395.2	336.0	277.5	243.7	225.2	213.6	208.9	217.8	212.7
Kwara	487.9	437.7	383.4	327.0	300.1	287.2	284.7	276.5	279.1	272.8
Lagos	392.6	314.4	243.4	186.2	167.4	180.9	221.4	257.7	283.7	274.6
Nasarawa	393.0	377.6	357.3	349.8	332.7	311.3	266.6	228.5	224.4	217.8
Niger	449.2	413.3	385.9	365.7	342.7	318.6	276.1	238.4	229.5	225.7
Ogun	433.1	351.3	294.1	239.6	221.2	230.9	253.9	267.9	281.1	273.4
Ondo	488.3	439.3	401.9	351.4	323.3	306.2	291.6	272.8	266.4	259.9
Osun	524.6	478.0	439.1	389.9	363.3	346.1	333.0	319.5	320.9	311.8
Oyo	515.9	457.1	411.8	359.1	340.4	332.4	331.8	321.7	321.1	312.6
Plateau	314.2	297.8	282.7	285.8	272.1	244.7	198.7	165.7	158.0	152.7
Rivers	413.1	332.2	275.2	242.9	234.3	241.6	251.9	258.3	279.5	268.9
Sokoto	612.8	666.9	683.0	671.1	647.0	593.4	461.7	335.5	255.1	250.9
Taraba	386.0	368.9	342.8	350.2	351.1	345.5	311.2	267.5	254.1	248.6
Yobe	432.5	408.2	365.8	326.5	293.4	254.1	204.0	167.0	158.1	156.3
Zamfara	723.6	736.4	715.2	375.9	642.0	572.9	413.4	283.7	231.5	227.9

Southern states, which generally began the period with lower malaria mortality rates, largely maintained this advantage between 2010 and 2019. Anambra, Bayelsa, Delta, and Lagos showed only modest declines over the past decade. Bayelsa reduced mortality from 279.8 to 177.5 deaths per 100,000; Delta declined from 328.8 to 192.9; and Lagos gradually decreased from 392.6 to 274.6 deaths per 100,000. Anambra State maintained mortality rates between 212.0 and 319.5, with only slight fluctuations.

2. States with substantial improvements

Several high-burden states have demonstrated marked reductions in malaria mortality. For instance, Jigawa State reduced mortality from 546.4 to 152.9 deaths per 100,000, whereas Cross River declined steadily from 440.2 to 242.9 over the same period. These trends indicate significant progress in malaria control in some of the most severely affected countries.

3. States with fluctuating trends

Some states exhibited inconsistent trends, with fluctuating mortality rates over the decade. Imo State, for example, declined from 474.3 deaths per 100,000 in 2010 to 288.8 in 2014, before rising slightly to 292.6 in 2019. Similarly, Akwa Ibom decreased from 477.9 in 2010 to 287.6 in 2014 and then increased to 313.5 in 2019. Abia and Benue also experienced fluctuations, with periods of decline followed by minor increases. These non-linear trends suggest that while progress was made in some years, the gains were not consistently sustained throughout the decade.

### Inequality in malaria incidence and mortality

To quantify the extent of subnational disparity, summary inequality measures were calculated, including the Coefficient of Variation (COV), difference (D), ratio (R), Population Attributable Risk (PAR), and Population Attributable Fraction (PAF). For malaria incidence, the COV rose from 26.3% in 2010 to 46.3% in 2013 before declining to 28.1% in 2019, indicating a temporary worsening of inequality, followed by modest convergence (Table 3). The absolute difference in incidence between the highest and lowest states (D) peaked at 148.4 pp in 2013 and declined to 67.9 pp in 2019. The incidence ratio (R) also declined from 10.6 in 2014 to 2.1 in 2019. PAR values remained negative across all years, indicating that several states consistently exceeded the national averages. The most extreme incidence of PAR occurred in 2013 (− 65.8), whereas the least occurred in 2019 (− 38.4). Correspondingly, the incidence of PAF dropped from − 80.5% in 2014 to − 53.4% in 2019, showing a relative narrowing of the inequalities.

**Table 3:** Inequality in malaria incidence (subnational states)

Year	COV (%)	D	R	PAR	PAF (%)
2010	26.3	113.5	3.6	− 58.0	− 56.9
2011	32.3	126.5	4.6	− 59.6	− 63.2
2012	40.6	141.4	7.05	− 64.1	− 73.3
2013	46.3	148.4	9.9	− 65.8	− 79.9
2014	45.8	146.7	10.6	− 63.0	− 80.5
2015	39.2	131.2	8.1	− 54.1	− 74.6
2016	33.2	102.9	4.5	− 39.1	− 57.1
2017	32.9	90.4	4.8	− 45.7	− 65.5
2018	28.2	68.2	3.0	− 38.6	− 53.6
2019	28.1	67.9	3.0	− 38.4	− 53.4

COV (%) – Coefficient of Variation; measures relative variability in malaria incidence across subnational states. Higher values indicate greater inequality; D – Absolute difference in malaria incidence between the highest- and lowest-burden states; R – Relative ratio of malaria incidence between the highest- and lowest-burden states; PAR – Population Attributable Risk; indicates the absolute reduction in subnational incidence that would occur if all states had the same incidence as the reference (lowest-burden) state. Negative values reflect that the subnational average is lower than the reference group, or that reductions are measured relative to the highest-burden state; PAF (%) – Population Attributable Fraction; the proportion (%) of total malaria incidence attributable to subnational inequalities. Negative values occur because reductions are calculated relative to the reference state, indicating the fraction of incidence above the reference level

For malaria mortality, the COV increased from 24.0% in 2010 to 42.9% in 2013 and then reduced to 22.3% in 2019. The absolute difference (D) between the highest and lowest mortality states reached a maximum of 616.7 deaths per 1000 cases in 2013 and reduced to 162.9 by 2019. The mortality ratio (R) decreased from 4.3 in 2013 to 2.1 in 2019. The mortality PAR was highest in 2013 (− 211.4) and lowest in 2019 (− 77.3), indicating a consistent excess mortality above the national mean among high-burden states (Table 4). The PAF for mortality followed a similar trend, decreasing from − 42.9% in 2010 to − 33.6% in 2019, highlighting the gradual progress in reducing geographic disparities in malaria-related mortality. The findings show that although national malaria incidence and mortality have decreased over the past decade (2010–2019), substantial subnational disparities have persisted. The years between 2010 and 2014 represented the period of the highest burden across many states, particularly in Northern Nigeria. Between 2015 and 2019, most states recorded improvements in both the incidence and mortality indicators. Although substantial inequality in malaria incidence and mortality persists, a reduction in the magnitude of disparities across states was observed in 2019. This is reflected in the narrowing differences and ratios across states and the decline in the COV, PAR, and PAF values for both incidence and mortality.

**Table 4:** Inequality in malaria mortality (subnational states)

Year	COV (%)	D	R	PAR	PAF (%)
2010	24.0	460.1	2.6	− 211.0	− 42.9
2011	31.6	513.0	3.1	− 210.9	− 46.0
2012	38.2	559.9	3.4	− 192.6	− 45.2
2013	42.9	616.7	4.3	− 211.4	− 53.2
2014	41.6	553.7	4.3	− 202.1	− 54.7
2015	32.7	412.6	3.3	− 148.8	− 45.1
2016	23.8	270.9	2.4	− 81.2	− 29.9
2017	27.1	219.9	2.7	− 108.7	− 46.3
2018	22.5	172.3	2.1	− 82.8	− 35.0
2019	22.3	162.9	2.1	− 77.3	− 33.6

COV (%) – Coefficient of variation, measuring relative inequality in malaria mortality across states; higher values indicate greater disparity. Absolute difference between the highest and lowest region-specific mortality rates; R – Ratio of the highest to lowest state-specific mortality rates; PAR – Population Attributable Risk; the absolute reduction in national malaria mortality that would occur if all states had the same mortality rate as the reference (best-performing) region. Negative values indicate that the subnational states average is lower than the reference value, reflecting a reduction in mortality; PAF (%) – Population Attributable Fraction; the proportion of national malaria mortality that could be reduced if all states matched the reference region. Negative values represent relative reductions compared to the reference

## Discussion

Malaria remains a significant public health challenge in Nigeria, particularly among children under five years of age. Between 2010 and 2019, the national-level malaria incidence declined substantially across Nigeria, with most states recording reductions of approximately 30–50% in incidence cases per 1000 population and 35–60% in malaria mortality per 100,000 population; however, these national averages masked substantial subnational disparities. Northern states, such as Zamfara, Katsina, and Kano, consistently bore a disproportionately higher malaria burden, whereas southern states, including Lagos, Delta, and Anambra, maintained relatively low and stable malaria indicators throughout the same period. These disparities are driven by several structural determinants. The ecological conditions of the Sahel and savannah zones in the north may facilitate longer mosquito breeding periods [20]. Coupled with socioeconomic disadvantages, including poverty, low maternal education, and under-resourced healthcare systems, these factors are likely to be associated with higher vulnerability to malaria among children [21, 22]. Insecurity and conflict, particularly in the northeast, further disrupt healthcare delivery and hinder access to preventive measures [23]. The modest improvement in inequality indicators post-2015 coincided with the scaling up of malaria control efforts in Nigeria, including the distribution of insecticide-treated nets (ITNs), seasonal malaria chemoprevention (SMC), and expanded access to artemisinin-based combination therapies (ACTs). Evidence from regional studies, including those in neighboring contexts, suggests that such interventions may contribute to reductions in the malaria burden and child mortality [24, 25]. Nevertheless, the persistently high burden in some states by 2019 suggests that implementation gaps driven by supply chain failures, limited community engagement, and weak health information systems continue to impede equitable progress [8].

Nigeria’s experience illustrates the broader challenges faced by the continent. Despite sustained national strategies, the vast population, ecological diversity, and entrenched inequalities of the country complicate malaria elimination. Although countries such as Egypt, Cape Verde, and Algeria have advanced toward malaria-free certification through integrated and sustained interventions, these lessons are context-specific; demographic, ecological, and health system differences mean that strategies successful elsewhere may require substantial adaptation to be effective in Nigeria [26, 27]. Cape Verde’s progress, for example, stems from strict importation controls, targeted vector elimination, and strong community awareness initiatives, elements that Nigeria struggles to replicate [28]. Northern Nigerian states continue to face severe stockouts of essential commodities and fragile data systems, which impede the effective management of severe malaria cases and overall health service delivery, contrasting sharply with the coordinated malaria control efforts in countries that have achieved substantial reductions in the malaria burden [13]. Rwanda and Eswatini offer additional lessons. Rwanda’s success stems from strong community health worker (CHW) programs and real-time digital surveillance, which enabled rapid responses to outbreaks [29]. Eswatini, although not malaria-free, has significantly reduced transmission through case investigation and widespread rapid testing [30]. Both countries underscore the importance of locally tailored strategies, sustained political commitment and decentralized health systems. Drawing from these examples, Nigeria could strengthen malaria control by expanding CHW-led surveillance, improving supply chain management, and implementing region-specific vector control and community engagement strategies adapted to its ecological and systemic contexts [31].

Nigeria exhibits notable subnational disparities in the malaria burden among children aged 0–4 years, with some states consistently experiencing higher incidence and mortality than others. The northern states, characterized by savanna ecology and seasonal rainfall patterns, remain conducive to malaria transmission. In addition, where documented, substandard housing structures may contribute to increased exposure to malaria [3, 32]. Compounding these barriers, such as poverty, poor maternal education, and limited access to care, reduces the likelihood of early diagnosis and treatment. While similar challenges have been observed in other settings [33], Nigeria-specific evidence supports the role of socioeconomic and health system factors in malaria outcomes [34]. Some southern states, particularly more urbanized ones, such as Lagos, may benefit from relatively stronger health infrastructure and more consistent public investment in malaria control measures. However, substantial heterogeneity exists, with other southern states (e.g., Imo, Abia, and Cross River) experiencing commodity stockouts, weak surveillance, and workforce limitations [35]. The observed subnational disparities in malaria incidence and mortality are likely influenced by multiple factors, including health system capacity, timing and coverage of interventions such as ITN distribution, and insecurity in the northeast, which may limit access to care [36]. These patterns are described based on the currently available evidence, as comprehensive data on the regional allocation of malaria funding are limited [35]. Security challenges in northeastern Nigeria have limited the reach of health services and disrupted surveillance systems and supply chains [8]. However, following the launch of the 2016–2020 National Malaria Strategic Plan (NMSP), improvements in equity and access began to emerge, suggesting that these targeted strategies were reaching previously neglected areas [15, 37]. Erratic year-to-year fluctuations in malaria incidence and mortality have been observed in some southern Nigerian states, including Imo, Akwa Ibom, and Abia, contributing to changes in subnational inequality metrics such as the PAR, PAF, and COV [38]. These fluctuations may reflect inconsistent program implementation, commodity shortages, or weak health surveillance [38]. Addressing these issues requires sustained oversight, stronger data systems, and subnational strategies that respond to local contexts.

This study drew on subnational malaria estimates derived from established data sources (IHME models) to assess spatial and temporal disparities. The decade-long trend analysis provides a comprehensive overview of malaria patterns. However, reliance on modelled data and the underlying primary survey sources presents limitations, particularly in conflict-affected zones with sparse reports. In addition, nationally aggregated annual incidence and mortality estimates corresponding to subnational data were not available through the WHO HEAT platform, limiting the direct benchmarking of state-level patterns against national averages**.** The WHO HEAT toolkit used for the analysis is primarily descriptive and does not support multivariate adjustment or causal inference, which should be considered when interpreting the findings. The analysis was based on malaria incidence and mortality estimates from 2010 to 2019, which may not fully reflect more recent changes in transmission dynamics, intervention coverage, or geopolitical conditions since 2020.

**Fig. 3:**
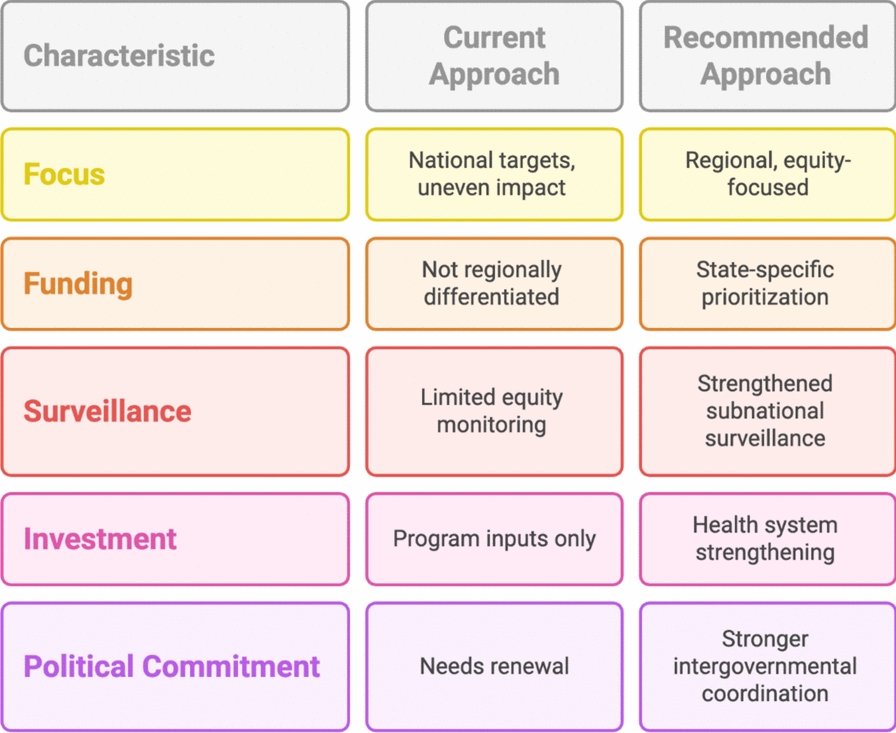
Comparison of current and recommended approaches to malaria control in Nigeria

This timeframe was selected because it represents the most recent period for which consistent and high-resolution subnational estimates were available at the time of analysis. Moreover, the study does not fully account for determinants such as household income or climate variability, not as an oversight, but because such variables are not consistently available at comparable subnational resolutions across the entire study period. Finally, as HEAT is designed primarily as a descriptive inequality-monitoring tool, the analysis is limited to identifying patterns and magnitudes of inequality and does not allow for multivariate adjustment, assessment of confounding factors, or causal inference regarding the effectiveness of specific interventions. Despite these limitations, this study underscores the critical need for subnational tailoring of malaria control strategies, and a shift away from a one-size-fits-all approach is imperative. Policymakers and partners must prioritize high-burden states for intensified intervention, ensuring better delivery of ACTs, ITNs, and SMC, particularly for vulnerable populations such as young children (Fig. 3) [23, 39]. This demands not only increased funding but also the implementation of structural reforms. Decentralized funding mechanisms and state-level autonomy can enhance program responsiveness and reduce delays. Funding decisions should be informed by real-time data and inequality indicators to ensure that the most vulnerable populations are prioritized. Improved surveillance and routine monitoring disaggregated at the state and local levels will be key to identifying emerging hotspots and addressing gaps in program delivery (Fig. 3). Alegana et al. (2016) demonstrated that reductions in malaria are often geographically uneven in Namibia, reinforcing the need for disaggregated data to guide responsive interventions [40]. Large-scale modeling studies echo this, showing that progress in high-burden areas often follows intensified and localized intervention efforts [41, 42].

## Conclusion

Nigeria has made important strides in reducing malaria incidence and mortality among children under five years of age nationally; however, the persistence of stark subnational inequalities, particularly between the northern and southern regions, continues to threaten child health outcomes and national malaria goals. While interpretations are constrained by the absence of nationally aggregated annual estimates alongside state-level data, the observed disparities remain substantial and are policy-relevant. Achieving malaria elimination in Nigeria requires urgent investment in equity-focused surveillance, strengthened health systems, and sustained political commitment to ensure real-time data availability, targeted resource allocation, and equitable access to life-saving interventions for all children, particularly in high-burden regions.

## Data Availability

The data used in this study were derived from the World Health Organization’s Health Equity Assessment Toolkit (HEAT), an open-access software application available at https://www.who.int/data/gho/health-equity. The HEAT software contains publicly available, preloaded data from the WHO Health Equity Monitor database. All analyses and interpretations in this study are solely those of the authors and do not necessarily reflect the views or policies of the WHO. Additional processed data and analysis files generated during the study are available from the corresponding author upon reasonable request.

## Abbreviations

IHME: Institute for Health Metrics and Evaluation
HEAT: Health Equity Assessment Toolkit
COV: Coefficient of variation
D: Difference
R: Ratio
PAR: Population attributable risk
PAF: Population attributable fraction
WHO: World Health Organization
NMSP: National Malaria Strategic Plan
ITNs: Insecticide-treated nets
IPTp: Intermittent preventive treatment in pregnancy
ACTs: Artemisinin-based combination
NMIS: Nigeria Malaria Indicator Survey
FCT: Federal Capital Territory
DHS: Demographic and Health Survey
STROBE: Strengthening the Reporting of Observational Studies in Epidemiology
MIS: Malaria Indicator Survey
LMIC: Low and Middle-Income Countries
SDH: Social Determinants of Health
HBHI: High burden to high impact
